# Fertility intention and its determinants of childbearing-age population in China after the three-child policy: a systematic review and meta-analysis

**DOI:** 10.7189/jogh.16.04074

**Published:** 2026-03-06

**Authors:** Jie Ren, Xiaonan Zhou, Yaozong Zheng, Xiaohan Ye, Lanyan Hu, Xiaochang Ma, Bin Lin, Lining Yang

**Affiliations:** 1 School of Public Health and Management, Guangzhou University of Chinese Medicine, Guangzhou, China; 2 China Joint Graduate School of Traditional Chinese Medicine, Suzhou, China; 3 First Clinical Medical College, Guangzhou University of Chinese Medicine, Guangzhou, China; 4 The Second Clinical Medical College, Guangzhou University of Chinese Medicine, Guangzhou, China; 5 Department of Cardiovascular Medicine, Xiyuan Hospital, China Academy of Chinese Medical Sciences, Beijing, China; 6 Second Affiliated Hospital of Shandong University of Traditional Chinese Medicine, Ophthalmology Department, Jinan, China

## Abstract

**Background:**

China’s sustained fertility decline has intensified population contraction and ageing. Following the rollout of the three-child policy, clarifying fertility intentions among the childbearing-age population and their determinants is crucial for effective policy design and fertility promotion.

**Methods:**

We conducted a systematic search of PubMed, Embase, the Cochrane Library, Web of Science, China National Knowledge Infrastructure, Wanfang, China Science and Technology Journal Database, and Chinese Biomedical Literature Database. We included in the analysis studies published from 31 May 2021 to 10 May 2025 that examined third-child fertility intention (TCFI) and its determinants among the childbearing-age population in China. We used the Joanna Briggs Institute Critical Appraisal Checklist for cross-sectional studies to assess the quality of studies. Further, we estimated pooled prevalence with random-effects models.

**Results:**

A total of 37 studies were included in the analysis (n = 113 009 participants). The pooled prevalence of TCFI and willingness to have another child (WHAC) were 7.6% and 12.7%. Factors associated with higher TCFI included age >35 years, male sex, education at or below high school level, monthly personal income >CNY 2000, ethnic minority status, having at least one non-only-child spouse, and having no pension insurance. For WHAC, remarriage and good self-assessed health were significant determinants.

**Conclusions:**

Both TCFI and WHAC remain low in China’s childbearing-age population. Enhancing childcare subsidies, strengthening medical security, and addressing core economic constraints such as housing and education may help raise fertility intentions.

**Registration:**

PROSPERO: CRD420251036086.

Low fertility has become a worldwide issue. Persistently low fertility, together with population contraction and ageing, will pose serious economic challenges and increase pressure on health systems, social security programs, and the labour force [1]. According to the United Nations World Population Prospects 2024, more than half of countries and regions worldwide have fertility below the replacement level of 2.1 live births per woman; nearly one-fifth of countries and regions worldwide, including China, are experiencing ‘ultra-low’ fertility, with a lifetime average of fewer than 1.4 live births per woman [2]. China’s annual births have declined for seven consecutive years since 2017; although there was a slight rebound in 2024, the crude birth rate in 2024 was only 6.77‰, and the natural growth rate was −0.99‰ [3]. China has recorded negative population growth for three consecutive years, and the demographic outlook remains concerning. Thus, the three-child policy promulgated in 2021 has not lifted China out of low fertility. Fertility intention is regarded as a predictor of fertility behaviour. Numerous surveys under the three-child policy have assessed fertility intentions among China’s childbearing-age population through nationwide large-sample and local studies, covering women of childbearing age, university students, teachers, nurses, and civil servants [4–11].

Fertility intention is shaped by multiple factors. According to the State of World Population 2025 by the United Nations Population Fund, declining fertility rates are mainly driven by economic and social barriers, including economic instability, gender discrimination, lack of support from partners and communities, inadequate sexual and reproductive health services, and insufficient childcare and education services, rather than by a simple erosion of fertility intentions [12]. These findings are consistent with results from two survey studies conducted in China, which show that economic costs and gender discrimination are key factors contributing to the gap between fertility intentions and actual fertility behaviour among individuals of reproductive age [13,14]. Several cross-sectional studies suggest that expanding social security and family support, increasing the supply of inclusive childcare services, improving maternal and child healthcare, and promoting gender equality can enhance fertility intentions [15–17].

Current evidence on fertility intentions under the three-child policy is dominated by local surveys, yielding heterogeneous results across regions and leaving no unified conclusions or comprehensive, systematic evidence. Therefore, we conducted a systematic review and meta-analysis to synthesise fertility intentions among China’s childbearing-age population under the three-child policy and to analyse the multidimensional determinants of third-child fertility intention (TCFI), with the aim of informing more precise and effective fertility policies.

## METHODS

We conducted this systematic review and meta-analysis in accordance with the PRISMA guidelines (Table S1 in the **Online Supplementary Document**) [18]. We registered the study protocol prospectively in PROSPERO (CRD420251036086).

### Data sources and search strategy

We conducted a comprehensive search across multiple electronic databases, including PubMed, Embase, Cochrane Library, Web of Science, China National Knowledge Infrastructure (CNKI), Wanfang, China Science and Technology Journal Database (VIP), and Chinese Biomedical Literature Database (CBM). The English search terms included: fertility desire, fertility intention, fertility willingness, fertility motivation, fertility purpose, fertility plan, fertility need, reproductive intention, reproductive decision making, desire to have children, desire for child, childbearing desire, childbearing intention, intention to give birth, childbearing-age youth, childbearing-age women, childbearing-age group, childbearing-age population, childbearing age, reproductive age, youth, women, female, resident, adults, China, and Chinese. The search period spanned from 31 May 2021 to 10 May 2025 (Table S2 in the **Online Supplementary Document**).

### Inclusion and exclusion criteria

Studies were eligible for inclusion in this meta-analysis if they met the following inclusion criteria: study design (original cross-sectional studies), population (Chinese individuals of childbearing age), investigation period (conducted after 31 May 2021), reported the prevalence of TCFI, provided adequate quantitative data for analysis, published in Chinese or English, and were of moderate or high quality. Exclusion criteria referred to: duplicate publications, reviews, case reports, qualitative studies, or conference abstracts, unavailable full text, insufficient data for analysis, duplicate data sets from the same research source, non-Chinese or non-English literature, and low-quality studies.

### Study screening

For reference management and duplicate removal, we imported all retrieved records into NoteExpress, version 4.1.0.10030 (Beijing Aegean Software Ltd, Beijing, China). Two reviewers (JR and XZ) independently screened the titles and abstracts, followed by full-text evaluation to determine final inclusion. Any disagreements were resolved through discussion or consultation with a third reviewer (LY).

### Outcome measures

Fertility desire refers to a general subjective aspiration for having children without specific quantitative or conditional constraints [19,20]. Fertility behaviour denotes the actual reproductive actions undertaken by individuals. In contrast, fertility intention is a multidimensional concept encompassing an individual’s or family’s desired number of children, gender preferences, desired childbearing age, and preferred birth interval [16,21]. Notably, fertility intention is widely recognised as a primary predictor of fertility behaviour [21,22]. In this study, we focused on the dimension of the number of children in fertility intention, namely the intention to have a third child. Therefore, we analysed two primary outcomes. The first was the TCFI prevalence, defined as the percentage of participants willing to have three or more children, calculated by dividing the number of respondents expressing such willingness by the total sample and multiplying by 100. The second was the willingness to have another child (WHAC) among individuals with two children, calculated by dividing the number of respondents with such intention by the total sample and multiplying by 100. We used 2 × 2 contingency tables to extract raw data on the influencing factors from the included studies and to calculate odds ratios (ORs).

### Operationalisation and standardisation strategies

For the TCFI analysis, we restricted the study population to childbearing-age individuals, without accounting for variation in their actual fertility behaviours. TCFI is primarily characterised by two outcome indicators – the desired number of children and willingness to have a third child. For the WHAC analysis, we limited the study population to individuals with two children, and WHAC was assessed using two outcome indicators – willingness to have an additional child and willingness to have a third child. Notably, in the studies included in the TCFI analysis, if the number of participants with two children could be identified, this subset was extracted and integrated separately into the WHAC analysis. Considering the differences in survey instruments and question frameworks across the original studies, we adopted a standardisation strategy for the two outcomes – TCFI and WHAC. We classified individuals with a desired number of children ≥3 or who reported willingness to have a third child as having TCFI, and those with a desired number of children <3 or who reported unwillingness or uncertainty about having a third child as not having TCFI. Among parents who already had two children, we classified those who reported willingness to have one more child or to have a third child as having WHAC. We classified all other participants as not having WHAC.

### Quality assessment

Two reviewers (JR and XZ) independently appraised the study quality using the Joanna Briggs Institute (JBI) Critical Appraisal Checklist for cross-sectional studies. This checklist comprises eight core items. Studies are categorised into three quality levels based on total scores: high quality (7–8 points), moderate quality (4–6 points), and low quality (≤3 points). We included in the final analysis only studies that met the ‘moderate or above’ quality criterion. Discrepancies were resolved through discussion with LY.

### Data extraction

Two reviewers (XZ and YZ) independently extracted data into standardised Microsoft Excel 2019 (Microsoft Corporation, Redmond, Washington, USA) forms, including author name, publication year, study region, target population, sample size, number and proportion of respondents expressing willingness to have three or more children, number of respondents with two children, and proportion expressing willingness for another birth. Any inconsistencies were resolved through face-to-face discussion.

### Statistical analysis

We generated descriptive summaries to present study characteristics and major outcomes. We analysed data using Stata, version 18.0 (StataCorp LLC, College Station, Texas, USA). Further, we employed a random-effects model to estimate pooled effect sizes. We reported ORs with 95% confidence intervals (CIs) for principal outcomes and associated variables. Moreover, we generated Forest plots to visualise the meta-analysis results. We assessed the heterogeneity across studies using Cochran’s Q test and the *I^2^* statistic. *I^2^*<25% indicated no heterogeneity, while values of 25–50%, 50–75%, and ≥75% represented low, moderate, and high heterogeneity, respectively. To explore potential sources of heterogeneity, we conducted subgroup analyses, and sensitivity analyses were performed by sequentially omitting individual studies to assess the robustness of the pooled estimates. We examined publication bias with Egger’s regression test, and *P* < 0.05 indicated significant bias.

## RESULTS

### Study selection

**Figure 1:**
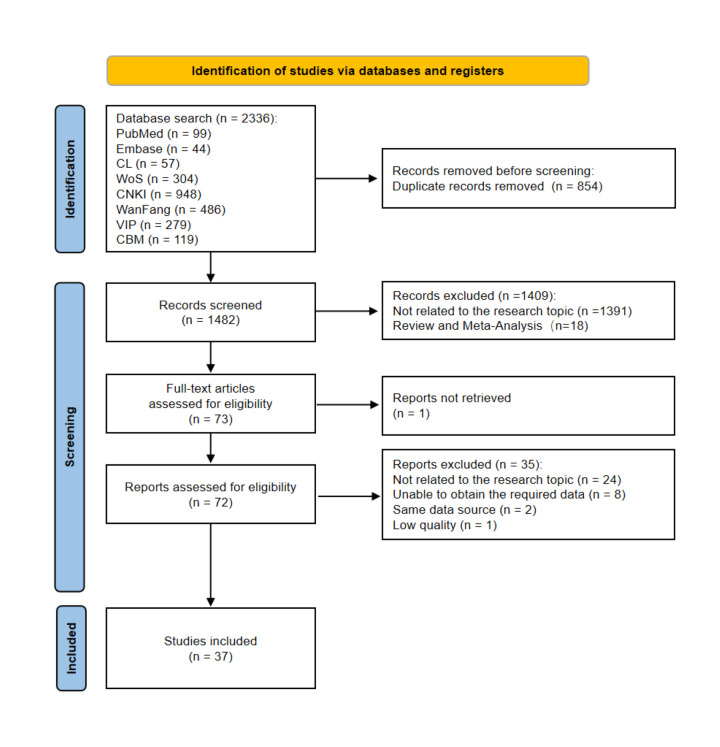
The PRISMA flowchart for the selection of studies.

We retrieved a total of 2336 records from electronic databases, including PubMed (n = 99), Embase (n = 44), Web of Science (n = 304), Cochrane Library (n = 57), CNKI (n = 948), Wanfang (n = 486), VIP (n = 279), and CBM (n = 119). After removing 854 duplicates, we excluded 1409 articles based on title and abstract screening for irrelevance. One study was excluded due to an inaccessible full text. Following a full-text assessment of 72 articles, we excluded 35 studies for not meeting the inclusion criteria. Ultimately, 37 studies were included in the systematic review and meta-analysis (**Figure 1**).

### Study characteristics

This meta-analysis incorporated 37 cross-sectional studies published between 2021 and 2025 (**Table 1**). Together, these studies involved 113 009 participants, with individual sample sizes ranging from 205 to 15 332. Most studies collected data through self-administered questionnaires. Reported prevalence of TCFI ranged from 1.10–53.60%, and WHAC ranged from 2.04–54.23%. All included studies were of medium or high quality according to the JBI checklist; specifically, seven were of medium quality and 30 of high quality. One low-quality study was excluded.

**Table 1:** Characteristics of included studies

Study	Region	Population	Sample size, n	TCFI, n	TCFI rate, %	People with two children, n	WHAC, n	WHAC rate, %
Zhu *et al.*, 2023 [23]	Beijing	Women of childbearing age	379	26	6.86	NA	NA	NA
Zheng *et al.*, 2025 [16]	Chongqing	Women of childbearing age	9717	NA	NA	3960	81	2.04
Zhang *et al.*, 2022 [24]	Northwest China	People of childbearing age	1610	303	18.82	NA	NA	NA
Yu CY *et al.*, 2024 [25]	China	Highly educated people of childbearing age	1242	NA	NA	1242	175	14.09
Yu DD *et al.*, 2024 [26]	Qiqihaer	People of childbearing age	230	40	17.39	NA	NA	NA
Xiao, 2023 [27]	DD District, Hubei	Employed women of childbearing age	205	20	9.76	59	10	16.95
Wu, 2023 [28]	N City, Jiangxi	Employed women of childbearing age	405	6	1.48	NA	NA	NA
Wang *et al.*, 2023 [29]	Wuhan	Women with two children	1756	NA	NA	1756	382	21.75
Sun, 2022 [30]	Western Hunan	Women of childbearing age	500	268	53.60	NA	NA	NA
Song *et al.*, 2024 [31]	Hebei	Married women of childbearing age	1104	74	6.70	NA	NA	NA
Shen, 2023 [32]	Shandong	Women of childbearing age	13 278	550	4.14	NA	NA	NA
Qiu *et al.*, 2022 [33]	Hainan	Employed women with two children	868	NA	NA	868	80	9.22
Qian *et al.*, 2024 [9]	Chongqing	Nurses of childbearing age	509	9	1.77	NA	NA	NA
Pei, 2022 [34]	G City, Hebei	Women of childbearing age	745	78	10.47	NA	NA	NA
Liu JF *et al.*, 2024 [35]	Qinghai	Women of childbearing age	1441	43	2.98	NA	NA	NA
Liu *et al.*, 2022 [36]	Nanchong	People of childbearing age	265	3	1.10	NA	NA	NA
Liu SL *et al.*, 2024 [37]	China	People of childbearing age	1276	85	6.66	NA	NA	NA
Lin *et al.*, 2024 [38]	Greater Bay Area	Residents of childbearing age	3556	371	10.43	1053	160	15.19
Liao *et al.*, 2025 [39]	Yunnan	Young mothers	3092	NA	NA	1605	66	4.11
Liao, 2023 [40]	Gansu	Perinatal women	3070	1087	35.41	NA	NA	NA
Li, 2022 [41]	Liaoning	Women with two children	465	NA	NA	465	103	22.15
Kuang *et al.*, 2023 [42]	Western Hunan	Women of childbearing age	543	129	23.76	142	77	54.23
He, 2024 [43]	Xiangtan	Women of childbearing age with at least one child	507	12	2.37	NA	NA	NA
Gong *et al.*, 2023 [44]	Changzhou	Women of childbearing age	4426	105	2.37	882	38	4.31
Gao *et al.*, 2023 [45]	Xi'an	Married women with two children	3250	NA	NA	3250	265	8.15
Zhu *et al.*, 2022 [46]	Shanghai	Couples with one or two children	1026	33	3.22	224	21	9.38
Zhang *et al.*, 2025 [47]	China	Clinicians	698	174	24.93	NA	NA	NA
Zhang *et al.*, 2022 [48]	Mainland China	University students	6680	180	2.70	NA	NA	NA
Yue *et al.*, 2023 [49]	China	People of childbearing age	2115	642	30.35	819	240	29.30
Yang *et al.*, 2024 [50]	Guangdong	Mothers with two children	603	NA	NA	603	63	10.45
Yan *et al.*, 2021 [51]	Mainland China	People of childbearing age	15 332	1861	12.14	NA	NA	NA
Xu *et al.*, 2024 [52]	Hangzhou	Millennial parents with two children	520	NA	NA	520	105	20.19
Wang *et al.*, 2022 [53]	China	Kindergarten teachers and subjects from other occupations	1042	28	2.69	NA	NA	NA
Qiao YT *et al.*, 2024 [54]	Shandong, Hunan, and Yunnan	Women of childbearing age	8002	NA	NA	3051	345	11.31
Qiao P *et al.*, 2024 [55]	Nanjing	Female university students	1124	14	1.25	NA	NA	NA
Jing *et al.*, 2022 [21]	China	People of childbearing age	9243	1203	13.02	1419	277	19.52
Chen *et al.*, 2023 [22]	Central China	People of childbearing age	13 479	NA	NA	7056	750	10.63

NA – not available, TCFI – third-child fertility intention, WHAC – willingness to have another child

### Pooled prevalence

**Figure 2:**
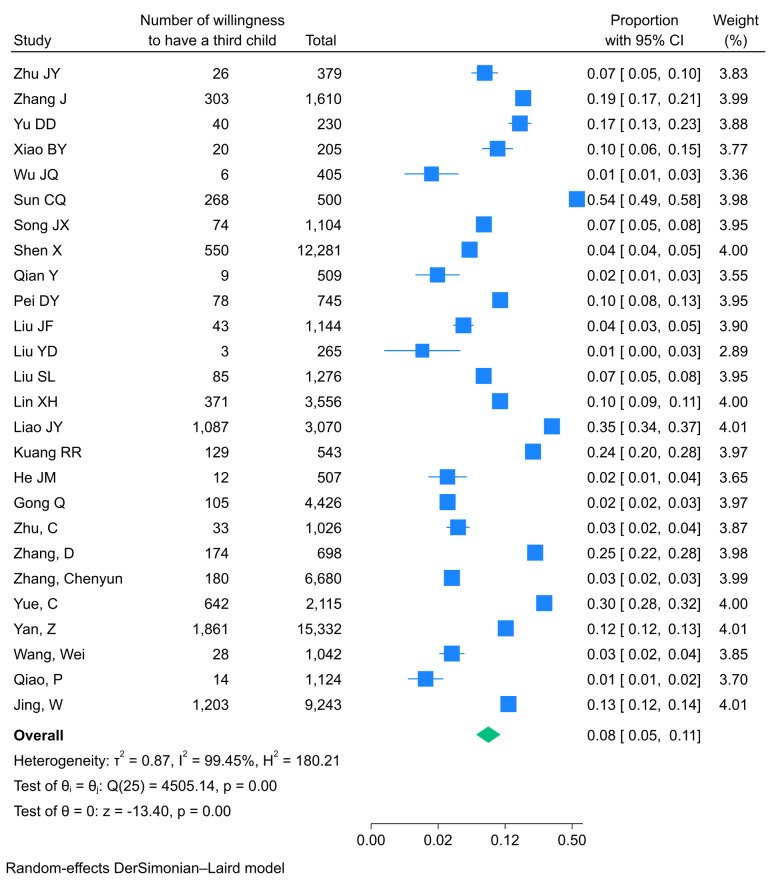
Forest plots of the pooled prevalence of TCFI. CI – confidence interval, TCFI – third-child fertility intention.

**Figure 3:**
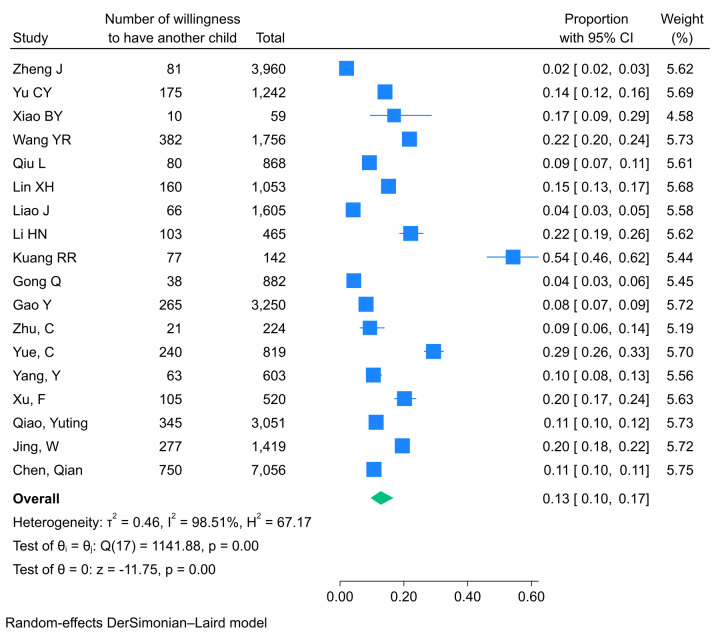
Forest plots of the pooled prevalence of WHAC. CI – confidence interval, WHAC – willingness to have another child.

The overall pooled TCFI prevalence was 7.6% (95% CI = 5.4–10.6%; *I^2^* = 99.45%, *P* < 0.001), while the pooled WHAC prevalence was 12.7% (95% CI = 9.6–16.8%; *I^2^* = 98.51%, *P* < 0.001). Given the substantial heterogeneity among studies, we applied random-effects models using the DerSimonian-Laird method to estimate the pooled prevalence. We generated Forest plots to visualise the outcomes (**Figure 2**, **Figure 3**). Based on these findings, subgroup and sensitivity analyses were further conducted.

### Subgroup analysis

To identify potential sources of heterogeneity, we stratified subgroup analyses by publication year, sample size, and region. For studies published before 2023 and in or after 2023, pooled TCFI prevalence was 8.4% (95% CI = 5.2–13.2) and 7.2% (95% CI = 4.2–12.1) (Figure S1 in the **Online Supplementary Document**). Corresponding WHAC prevalence was 14.3% (95% CI = 9.2–21.6) and 12.4% (95% CI = 8.8–17.1) (Figure S2 in the **Online Supplementary Document**). No statistically significant subgroup differences were detected (*P* = 0.67 and *P* = 0.59).

When grouped by sample size, studies with <2693 participants yielded a pooled TCFI prevalence of 7.3% (95% CI = 4.6–11.4), compared to 8.1% (95% CI = 4.4–14.6) in studies with larger samples (Figure S1 in the **Online Supplementary Document**). For WHAC, studies with <1610 participants reported a pooled prevalence of 14.7% (95% CI = 10.4–20.4), whereas those with ≥1610 participants had 8.7% (95% CI = 5.2–14.4) (Figure S2 in the **Online Supplementary Document**). Between-group differences were not significant (*P* = 0.78 and *P* = 0.09).

Subgroup analysis by region revealed the highest pooled TCFI prevalence in the Northeastern region, at 17% (95% CI = 13–23), while the lowest pooled prevalence was observed in the Eastern region, at 5% (95% CI = 3–7) (Figure S1 in the **Online Supplementary Document**). For WHAC, the highest pooled prevalence of 22% was reported in the Central (95% CI = 10–42) and Northeastern (95% CI = 19–26) regions, whereas the lowest prevalence was identified in the Western region, at 3% (95% CI = 1–6) (Figure S2 in the **Online Supplementary Document**). These regional differences were statistically significant (*P* < 0.001).

### Sensitivity analysis

We applied a leave-one-out sensitivity analysis to evaluate the influence of each study on the pooled estimates. Exclusion of any single study did not materially alter the combined TCFI or WHAC prevalence (Figures S3 and S4 in the **Online Supplementary Document**), indicating stability and robustness of the meta-analytic results.

### Publication bias

Egger’s regression test revealed no evidence of publication bias for either TCFI (*P* = 0.622) or WHAC (*P* = 0.560).

### Factors associated analysis

We performed random-effects modelling to examine the effects of demographic and socioeconomic variables, including age, sex, occupation, educational attainment, income, place of residence, marital status, ethnicity, number of children, only-child status, policy awareness, and insurance coverage, on fertility intentions. The analysis identified age, sex, education, income, ethnicity, only-child status, and pension insurance as significant predictors of TCFI (**Table 2**). Specifically, a meta-analysis of 10 studies showed that participants aged >35 years were approximately twice as likely to report TCFI as those aged ≤35 (OR = 1.968; 95% CI = 1.526–2.537). Furthermore, six studies indicated that men were more than twice as likely as women to report a willingness to have a third child (OR = 2.356; 95% CI = 1.550–3.582) and 11 studies demonstrated that respondents with a high-school education or lower were significantly more likely to report TCFI than those with higher education (OR = 2.274; 95% CI = 1.806–2.861). Moreover, three studies found that individuals with a monthly income exceeding CNY 2000 were more likely to have three children (OR = 1.661; 95% CI = 1.054–2.619), and seven studies revealed that ethnic minorities were more likely than Han participants to express TCFI (OR = 1.305; 95% CI = 1.058–1.608). Further, three studies reported that respondents with at least one non-only-child spouse had a higher intention for a third birth than those where both were only children (OR = 1.289; 95% CI = 1.113–1.492) and three additional studies found that individuals without pension insurance had higher TCFI than those covered by pension insurance (OR = 2.159; 95% CI = 1.407–3.312). No significant associations were observed for occupation, residence, marital status, number of existing children, policy awareness, or medical insurance status.

**Table 2:** Factors associated with TCFI

Variables	OR (95% CI)	Studies, n	*I^2^*	*P*-value
Aged >35 years	1.968 (1.526–2.537)*	10	83.69	0.0000
Male	2.356 (1.550–3.582)*	6	92.98	0.0001
Public official	1.010 (0.807–1.263)	7	67.75	0.9330
High school and below	2.274 (1.806–2.861)*	11	82.46	0.0000
Monthly personal income CNY > 2000	1.661 (1.054–2.619)*	3	0.00	0.0288
Rural	1.365 (0.877–2.124)	7	93.98	0.1682
Married	2.015 (0.966–4.204)	5	65.62	0.0619
Minority	1.305 (1.058–1.608)*	7	22.34	0.0128
With children	1.815 (0.727–4.529)	4	88.98	0.2014
At least one person is not an only child	1.289 (1.113–1.492)*	3	21.86	0.0007
Familiar with the policy	1.144 (0.661–1.979)	3	0.00	0.6306
Without pension insurance	2.159 (1.407–3.312)*	3	71.34	0.0004
With health insurance	0.510 (0.220–1.182)	4	91.54	0.1164

CI – confidence interval, OR – odds ratio, TCFI – third-child fertility intention

*Significantly associated factors.

For WHAC, random-effects analysis indicated that only marital status and self-assessed health were significant determinants (**Table 3**). Meta-analysis of three studies revealed that remarried individuals were more likely to plan another child than those in their first marriage (OR = 2.143; 95% CI = 1.186–3.874). Furthermore, two studies showed that participants reporting good health had higher WHAC than those reporting fair or poor health (OR = 1.341; 95% CI = 1.102–1.632). No significant differences were identified with respect to age, sex, gender composition of existing children, occupation, residence, ethnicity, income, or parental support.

**Table 3:** Factors associated with WHAC

Variables	OR (95% CI)	Studies, n	*I^2^*	*P*-value
Aged >35 years	0.777 (0.604–1.000)	7	76.54	0.0502
Male	0.863 (0.252–2.961)	2	83.78	0.8154
Two boys or two girls	0.959 (0.675–1.363)	7	89.26	0.8174
Public servants	0.841 (0.494–1.431)	4	81.24	0.5222
Rural	1.361 (0.911–2.034)	5	92.25	0.1326
Minority	1.537 (0.629–3.757)	5	96.11	0.3461
Per capita monthly household income CNY > 3000	2.496 (0.880–7.079)	2	96.08	0.0854
Remarriage	2.143 (1.186–3.874)*	3	86.44	0.0116
Good health status	1.341 (1.102–1.632)*	2	0.00	0.0034
With parental support	0.830 (0.657–1.048)	2	0.00	0.1169

CI – confidence interval, OR – odds ratio, WHAC – willingness to have another child

*Significantly associated factors.

## DISCUSSION

In this systematic review of 37 cross-sectional studies and 113 009 participants, we found that the pooled prevalence of TCFI among the childbearing-age population in China was 7.6%, whereas the pooled prevalence of WHAC was 12.7%. The meta-analysis indicated that age, sex, educational attainment, personal income, ethnicity, only-child status, and pension coverage were significant predictors of TCFI, whereas marital and health status were the main determinants of WHAC.

The overall prevalence of TCFI was 7.6%, consistent with previous national cross-sectional investigations in China [56,57], yet notably lower than that reported in several developed and developing countries. A survey conducted in Greece found that 29.0% of young adults desired three children [58], and a longitudinal study in Israel found that 38.7% of respondents expected to have three children [59]. In India, an investigation of 1979 married couples revealed that 14.5% preferred three children and 15.3% already had three [60]. These discrepancies likely stem from China’s comparatively higher financial and time costs of raising multiple children. The overall prevalence of WHAC was 12.7%, consistent with findings by Yang and colleagues [61], slightly exceeding the 9.6% reported by Ning and collaborators among two-child families [62], yet below the 20.2% identified by Xu and colleagues in millennial parents residing in eastern China [52]. This variation may be attributable to differences in age composition and regional distribution. In South Asia, approximately one-third (31.1%) of women with at least one child reported a desire to have another child, ranging from 19.4% in Nepal to 42.3% in Afghanistan [63]. This higher level of willingness among developing countries is often associated with the value placed on children as contributors to household labour and economic stability. Due to the high heterogeneity, the pooled estimates of TCFI and WHAC should be interpreted as general trends rather than definitive conclusions.

We identified extremely high statistical heterogeneity in this study, compromising the generalisability and reliability of the pooled prevalence estimates. Multi-dimensional subgroup analyses revealed significant regional disparities, confirming that regional variation constitutes an important source of heterogeneity. The highest TCFI prevalence (17%) was reported in Northeast China, whereas the lowest rate (5%) was observed in East China. For WHAC, prevalence was highest in Central and Northeastern China (22%) and lowest in Western China (3%). In a meta-analysis of second-child fertility intentions, the intention rate in Eastern China (44%) was higher than in Western China (40%), with the higher level of economic development in Eastern China considered a key contributing factor. This finding differs from the results of the present study, which may be attributed to regional disparities in economic development that influence fertility and educational costs. Having three children entails a multiplicative increase in costs, such that regions with higher economic development are more vulnerable to cost constraints [64]. Although Northeast China exhibited the highest TCFI, this result may not be representative of the region's overall level due to the limited number of studies included in this subgroup. WHAC in Western China (3%) was significantly lower than that in Eastern China (11%). Parents who have already had two children possess practical parenting experience, and their decision-making focuses on the marginal cost of having an additional child. Populations with higher socioeconomic status demonstrate stronger resilience to fertility-related risks and are more likely to translate their fertility intention into actual behaviour [64,65].

Regarding age, individuals aged >35 years had higher TCFI scores than their younger counterparts. This may be because older respondents are more likely to already have two children and thus consider having a third to complete family size. Ren’s 2024 meta-analysis also supports this observation, indicating that families with two or more children had higher fertility intentions than those with fewer [66]. Furthermore, as childbearing potential declines with age, individuals may feel a stronger urgency to pursue an additional birth [52,67].

Gender disparities were also evident. Men exhibited higher TCFI than women, a trend consistent with multiple previous reports [66,68,69]. This difference may derive from traditional Confucian beliefs that emphasise male lineage continuity and assign primary caregiving responsibilities to women, for whom the physical and temporal burdens of childbirth are substantial [70,71]. Similar gender-related patterns have been observed in both South Korea and Nigeria [72,73].

Educational attainment played a crucial role in shaping attitudes toward childbearing. Participants with a high-school education or lower were more willing to have a third child than those with higher degrees, a finding consistent with other Chinese research [57,66]. According to Addisalem and colleagues, education enhances women’s professional capacity and awareness of childbearing health and family planning, thereby leading to a preference for smaller families [74]. Likewise, a Nigerian study among individuals aged 15–24 without prior childbirth found that secondary or tertiary education was associated with a lower preference for large families compared with no formal education [75].

Economic capacity also significantly influenced fertility intentions. Participants with a monthly income exceeding CNY 2000 were more willing to have three children. This likely reflects the significant financial burden of childbearing and childrearing. Low-income families tend to prioritise essential living expenses and exhibit greater caution regarding additional births. Evidence from India and South Korea indicates that higher socioeconomic status is positively associated with fertility intentions [76,77]. Such findings suggest that financial stress is a shared constraint across many Asian countries. To address this issue, the Chinese government released the Childcare Subsidy Implementation Plan in July 2025, providing an annual allowance of CNY 3600 per child under the age of three, which has attracted widespread public attention [78].

Ethnic background was another influential factor. Participants from ethnic minority groups showed stronger TCFI than those of the Han ethnicity. Historically, family-planning policies have been more lenient toward minority populations, allowing two or more children, which may have fostered long-term acceptance of larger families. Additionally, cultural continuity, labour demands, limited social welfare, and educational disparities further contribute to ethnic differences in childbearing behaviour.

The results also revealed that couples in which at least one spouse was not an only child were more likely to consider having a third child, consistent with findings from prior studies [79,80]. This may reflect the influence of family background, as individuals raised with siblings often hold more favourable perceptions of large families and greater emotional identification with multi-child households.

Another key determinant was pension insurance coverage. Those without pension protection exhibited higher TCFI than individuals with such coverage, consistent with previous empirical analyses [79,81]. Prior research has indicated that raising additional children can deplete family resources and reduce long-term retirement savings, whereas future support from offspring remains uncertain [82]. In contrast, stable pension systems reduce dependence on children for economic security, thereby diminishing the incentive to have additional births.

Regarding WHAC, both health and marital status emerged as significant predictors. Participants who self-assessed their health as good exhibited higher WHAC, consistent with studies that identify good physical health as a positive indicator of fertility intention [69]. This association is more pronounced in women, as good health enhances their ability to cope with the physiological and psychological demands of pregnancy and childrearing [83]. Marital status also exerted a strong influence. Remarried individuals showed a markedly higher WHAC than those in their first marriage, in agreement with findings by Lu and colleagues [84]. In blended families, having a shared child is often seen as a way to strengthen emotional bonds and family unity.

This study provided a comprehensive analysis of fertility intentions among China’s childbearing-age population following the implementation of the three-child policy; however, several limitations should be acknowledged. First, among the 37 studies included, most were conducted in eastern and central China, and some focused on specific populations. This geographical and population concentration may limit the generalizability of the findings. Second, although standardised definitions were applied in the present analysis, the validity and reliability of the original measurement instruments varied across studies, which may have influenced the robustness of the results. Third, although all included original studies adjusted for confounding factors using multivariate logistic regression, heterogeneity in grouping criteria across studies rendered direct pooling of the adjusted ORs infeasible. Therefore, we extracted raw data to calculate ORs, a methodological approach that may overlook the potential effects of confounding factors. Additionally, given the limited number of included studies, it was not feasible to explore sources of heterogeneity through subgroup analysis; thus, the results should be interpreted with caution.

## CONCLUSIONS

This study concludes that the fertility intention to have a third child among China’s childbearing-age population remains low. Age, sex, educational attainment, individual income, ethnicity, only-child status, and pension coverage were identified as key determinants of willingness to have a third child. Although the WHAC was slightly higher, it remained relatively low. Personal health condition and marital status were major determinants of WHAC. To enhance fertility intentions among the childbearing-age population in China, governmental policies should further strengthen childcare subsidies to alleviate the economic burden of child-rearing. Given that individuals with two existing children demonstrated a higher likelihood of having a third child, policy measures should prioritise this group and remarried families. Special attention should be devoted to improving healthcare support for these populations to reduce fertility-related health risks. In addition, addressing fundamental socioeconomic barriers such as housing and education remains essential for promoting sustainable fertility intentions.

## Additional material


Online Supplementary Document
