# Supplementary material for: Fertility intention and its determinants of childbearing-age population in China after the three-child policy: a systematic review and meta-analysis
Source: J Glob Health. 2026 Mar 6;16:04074. doi: 10.7189/jogh.16.04074 (PMC12967243; doi:10.7189/jogh.16.04074)
Supplement: Online Supplementary Document [file jogh-16-04074-s001.pdf]

Supplement to: Ren J, Zhou X, Zheng Y, Ye X, Hu L, Ma X, Lin B, Yang L.Fertility Intention and Their Determinants of Childbearing-age Population in Chinaafter the Three-Child Policy: A Systematic Review and Meta-Analysis. J Glob Health.2026;16: 04074.

Table S1. PRISMA Checklist

Table S2. Search Strategy

Figure S1. Subgroup analysis of TCFI

Figure S2. Subgroup analysis of WHAC

Figure S3. Sensitivity analysis of TCFI

Figure S4. Sensitivity analysis of WHAC

Table S1. PRISMA Checklist

Section and Topic	Item #	Checklist item	Location where item is reported
**TITLE**			
Title	1	Identify the report as a systematic review.	1
**ABSTRACT**			
Abstract	2	See the PRISMA 2020 for Abstracts checklist.	1-2
**INTRODUCTION**			
Rationale	3	Describe the rationale for the review in the context of existing knowledge.	2-3
Objectives	4	Provide an explicit statement of the objective(s) or question(s) the review addresses.	3
**METHODS**			
Eligibility criteria	5	Specify the inclusion and exclusion criteria for the review and how studies were grouped for the syntheses.	4-5
Information sources	6	Specify all databases, registers, websites, organisations, reference lists and other sources searched or consulted to identify studies. Specify the date when each source was last searched or consulted.	4
Search strategy	7	Present the full search strategies for all databases, registers and websites, including any filters and limits used.	4, Supplementary Table S2.
Selection process	8	Specify the methods used to decide whether a study met the inclusion criteria of the review, including how many reviewers screened each record and each report retrieved, whether they worked independently, and if applicable, details of automation tools used in the process.	4
Data collection process	9	Specify the methods used to collect data from reports, including how many reviewers collected data from each report, whether they worked independently, any processes for obtaining or confirming data from study investigators, and if applicable, details of automation tools used in the process.	6

Section and Topic	Item #	Checklist item	Location where item is reported
Data items	10a	List and define all outcomes for which data were sought. Specify whether all results that were compatible with each outcome domain in each study were sought (e.g. for all measures, time points, analyses), and if not, the methods used to decide which results to collect.	5
	10b	List and define all other variables for which data were sought (e.g. participant and intervention characteristics, funding sources). Describe any assumptions made about any missing or unclear information.	6
Study risk of bias assessment	11	Specify the methods used to assess risk of bias in the included studies, including details of the tool(s) used, how many reviewers assessed each study and whether they worked independently, and if applicable, details of automation tools used in the process.	6
Effect measures	12	Specify for each outcome the effect measure(s) (e.g. risk ratio, mean difference) used in the synthesis or presentation of results.	5
Synthesis methods	13a	Describe the processes used to decide which studies were eligible for each synthesis (e.g. tabulating the study intervention characteristics and comparing against the planned groups for each synthesis (item #5)).	4，6
	13b	Describe any methods required to prepare the data for presentation or synthesis, such as handling of missing summary statistics, or data conversions.	6
	13c	Describe any methods used to tabulate or visually display results of individual studies and syntheses.	6-7
	13d	Describe any methods used to synthesize results and provide a rationale for the choice(s). If meta-analysis was performed, describe the model(s), method(s) to identify the presence and extent of statistical heterogeneity, and software package(s) used.	6-7
	13e	Describe any methods used to explore possible causes of heterogeneity among study results (e.g. subgroup analysis, meta-regression).	7
	13f	Describe any sensitivity analyses conducted to assess robustness of the synthesized results.	7
Reporting bias assessment	14	Describe any methods used to assess risk of bias due to missing results in a synthesis (arising from reporting biases).	7
Certainty	15	Describe any methods used to assess certainty (or confidence) in the body of evidence for an outcome.	NA

Section and Topic	Item #	Checklist item	Location where item is reported
assessment			
**RESULTS**			
Study selection	16a	Describe the results of the search and selection process, from the number of records identified in the search to the number of studies included in the review, ideally using a flow diagram.	7，Figure 1
	16b	Cite studies that might appear to meet the inclusion criteria, but which were excluded, and explain why they were excluded.	NA
Study characteristics	17	Cite each included study and present its characteristics.	7，Table 1
Risk of bias in studies	18	Present assessments of risk of bias for each included study.	9
Results of individual studies	19	For all outcomes, present, for each study: (a) summary statistics for each group (where appropriate) and (b) an effect estimate and its precision (e.g. confidence/credible interval), ideally using structured tables or plots.	7-8, Figure 2-3 9-10, Table 2-3
Results of syntheses	20a	For each synthesis, briefly summarise the characteristics and risk of bias among contributing studies.	7-9
	20b	Present results of all statistical syntheses conducted. If meta-analysis was done, present for each the summary estimate and its precision (e.g. confidence/credible interval) and measures of statistical heterogeneity. If comparing groups, describe the direction of the effect.	7-8, Figure 2-3 9-10, Table 2-3
	20c	Present results of all investigations of possible causes of heterogeneity among study results.	8，Supplementary Figure S1-S2
	20d	Present results of all sensitivity analyses conducted to assess the robustness of the synthesized results.	8-9，Supplementary

Section and Topic	Item #	Checklist item	Location where item is reported
			Figure S3- S4
Reporting biases	21	Present assessments of risk of bias due to missing results (arising from reporting biases) for each synthesis assessed.	9
Certainty of evidence	22	Present assessments of certainty (or confidence) in the body of evidence for each outcome assessed.	8-9
**DISCUSSION**			
Discussion	23a	Provide a general interpretation of the results in the context of other evidence.	10-14
	23b	Discuss any limitations of the evidence included in the review.	14
	23c	Discuss any limitations of the review processes used.	14
	23d	Discuss implications of the results for practice, policy, and future research.	14-15
**OTHER INFORMATION**			
Registration and protocol	24a	Provide registration information for the review, including register name and registration number, or state that the review was not registered.	2
	24b	Indicate where the review protocol can be accessed, or state that a protocol was not prepared.	3-6
	24c	Describe and explain any amendments to information provided at registration or in the protocol.	3-6
Support	25	Describe sources of financial or non-financial support for the review, and the role of the funders or sponsors in the review.	15
Competing interests	26	Declare any competing interests of review authors.	15
Availability of data, code and other materials	27	Report which of the following are publicly available and where they can be found: template data collection forms; data extracted from included studies; data used for all analyses; analytic code; any other materials used in the review.	15

Table S2. Search Strategy

Database	Search strategy		Results
PubMed	#1	(fertility desire[Title/Abstract]) OR (fertility intention[Title/Abstract]) OR (fertility willingness[Title/Abstract]) OR (fertility motivation[Title/Abstract]) OR (fertility purpose [Title/Abstract]) OR (fertility plan[Title/Abstract]) OR (fertility need[Title/Abstract]) OR (reproductive intention [Title/Abstract]) OR (reproductive decision making[Title/Abstract]) OR (desire to have children[Title/Abstract]) OR (desire for child[Title/Abstract]) OR (childbearing desire[Title/Abstract]) OR (childbearing intention[Title/Abstract]) OR (intention to give birth[Title/Abstract])	9056
	#2	(childbearing age youth[Title/Abstract]) OR (childbearing age women[Title/Abstract]) OR (childbearing age group [Title/Abstract]) OR (childbearing age population[Title/Abstract]) OR (childbearing age[Title/Abstract]) OR (reproductive age[Title/Abstract]) OR (youth[Title/Abstract]) OR (women[Title/Abstract]) OR (female[Title/Abstract]) OR (resident[Title/Abstract]) OR (adults[Title/Abstract])	3060673
	#3	(China[Title/Abstract]) OR (Chinese[Title/Abstract])	616029
	#4	#1 AND #2 AND #3	158
		Publication date: from 2021/5/31 - 2025/5/10; Article Language: English	99
Embase	#1	'fertility desire':ti,ab,kw OR 'fertility intention':ti,ab,kw OR 'fertility willingness':ti,ab,kw OR 'fertility motivation':ti,ab,kw OR 'fertility purpose':ti,ab,kw OR 'fertility plan':ti,ab,kw OR 'fertility need':ti,ab,kw OR 'reproductive intention':ti,ab,kw OR 'reproductive decision making':ti,ab,kw OR 'desire to have children':ti,ab,kw OR 'desire for child':ti,ab,kw OR 'childbearing desire':ti,ab,kw OR 'childbearing intention':ti,ab,kw OR 'intention to give birth':ti,ab,kw	2035
	#2	'childbearing age youth':ti,ab,kw OR 'childbearing age women':ti,ab,kw OR 'childbearing age group':ti,ab,kw OR 'childbearing age population':ti,ab,kw OR 'childbearing age':ti,ab,kw OR 'reproductive age':ti,ab,kw OR 'youth':ti,ab,kw OR 'women':ti,ab,kw OR 'female':ti,ab,kw OR 'resident':ti,ab,kw OR 'adults':ti,ab,kw	4375482
	#3	'China':ti,ab,kw OR 'Chinese':ti,ab,kw	726396
	#4	#1 AND #2 AND #3	80
		Publication years: 2021-2025; Languages: English; Publication types: Article	44
Cochrane L	#1	(fertility desire):ti,ab,kw OR (fertility intention):ti,ab,kw	2517

ibrary		OR (fertility willingness):ti,ab,kw OR (fertility motivation):ti,ab,kw OR (fertility purpose):ti,ab,kw OR (fertility plan):ti,ab,kw OR (fertility need):ti,ab,kw OR (reproductive intention):ti,ab,kw OR (reproductive decision making):ti,ab,kw OR (desire to have children):ti,ab,kw OR (desire for child):ti,ab,kw OR (childbearing desire):ti,ab,kw OR (childbearing intention):ti,ab,kw OR (intention to give birth):ti,ab,kw	
	#2	(childbearing age youth):ti,ab,kw OR (childbearing age women):ti,ab,kw OR (childbearing age group):ti,ab,kw OR (childbearing age population):ti,ab,kw OR (childbearing age):ti,ab,kw OR (reproductive age):ti,ab,kw OR (youth):ti,ab,kw OR (women):ti,ab,kw OR (female):ti,ab,kw OR (resident):ti,ab,kw OR (adults):ti,ab,kw	1127545
	#3	(China):ti,ab,kw OR (Chinese):ti,ab,kw	59754
	#4	#1 AND #2 AND #3	105
		Date: 2021-05-31 to 2025-05-10; Language: English	57
Web of Science		Search in: Web of Science Core Collection; Timespan: 2021-05-31 to 2025-05-10 (Publication Date)	
	#1	TS=((fertility desire) OR (fertility intention) OR (fertility willingness) OR (fertility motivation) OR (fertility purpose) OR (fertility plan) OR (fertility need) OR (reproductive intention) OR (reproductive decision making) OR (desire to have children) OR (desire for child) OR (childbearing desire) OR (childbearing intention) OR (intention to give birth))	11656
	#2	TS=((childbearing age youth) OR (childbearing age women) OR (childbearing age group) OR (childbearing age population) OR (childbearing age) OR (reproductive age) OR (youth) OR (women) OR (female) OR (resident) OR (adults))	885327
	#3	TS=((China) OR (Chinese))	391470
	#4	#1 AND #2 AND #3	344
		Document Types: Article; Languages: English	304
CNKI		TKA=(育龄女性 + 育龄妇女 + 育龄人群 + 育龄群体 + 育龄青年 + 居民 + 青年) AND TKA=(生育意愿 + 生育计划 + 生育动机 + 生育意向)	2024
		时间范围：2021-05-31 至 2025-05-10	948
Wanfang		(题名或关键词:("育龄女性" OR "育龄妇女" OR "育龄人群" OR "育龄群体" OR "育龄青年" OR "居民" OR "青年")) AND (题名或关键词:("生育意愿" OR "生育计划" OR "生育动机" OR "生育意向"))	1039
		发表时间：2021 年至今	486
VIP		M=("育龄女性" OR "育龄妇女" OR "育龄人群" OR "	2127

		育龄群体" OR "育龄青年" OR "居民" OR "青年") AND M=("生育意愿" OR "生育计划" OR "生育动机" OR "生育意向")	
		时间范围：2021 至 2025	279
CBM	#1	"育龄女性"[常用字段:智能] OR "育龄妇女"[常用字段:智能] OR "育龄人群"[常用字段:智能] OR "育龄群体"[常用字段:智能] OR "育龄青年"[常用字段:智能] OR "居民"[常用字段:智能] OR "青年"[常用字段:智能]	156424
	#2	"生育意愿"[常用字段:智能] OR "生育计划"[常用字段:智能] OR "生育动机"[常用字段:智能] OR "生育意向"[常用字段:智能]	1075
	#3	#1 AND #2	282
		年代：2021 年至今	119

Figure S1. Subgroup analysis of TCFI

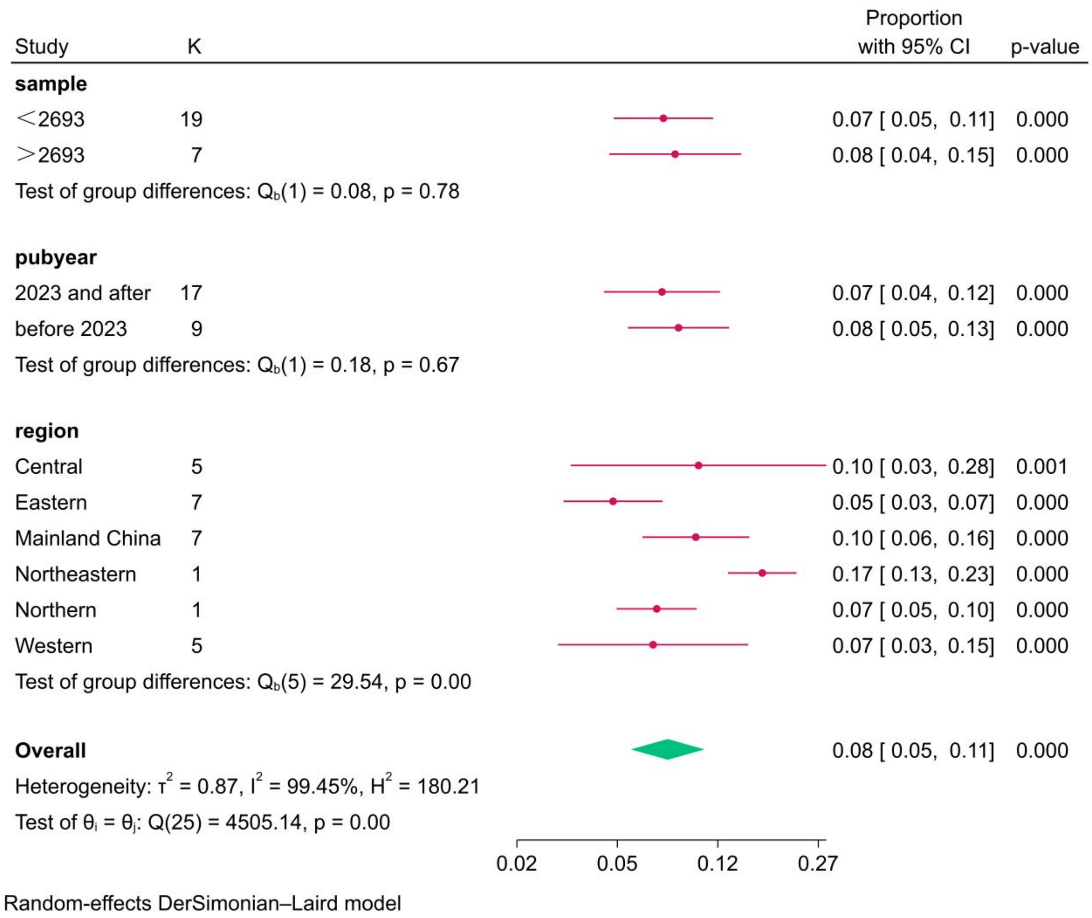

CI – confidence interval.

Figure S2. Subgroup analysis of WHAC

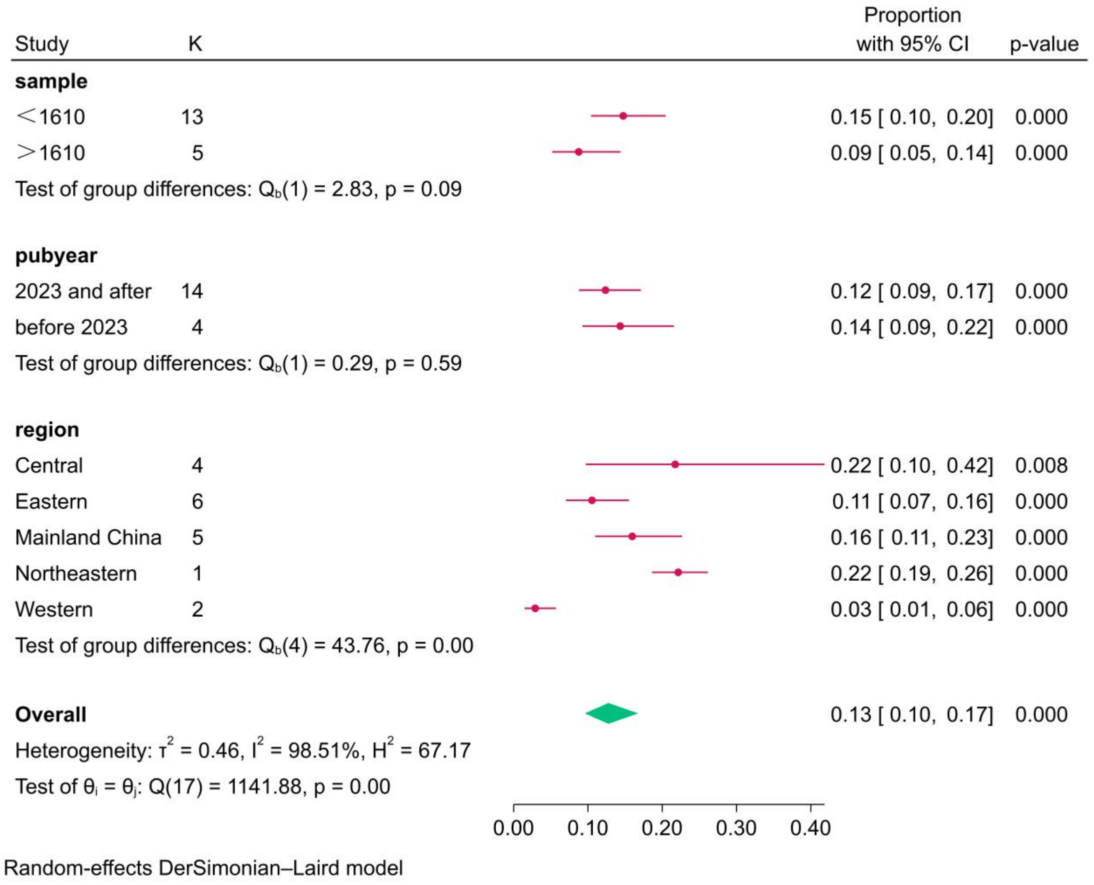

CI – confidence interval.

Figure S3. Sensitivity analysis of TCFI

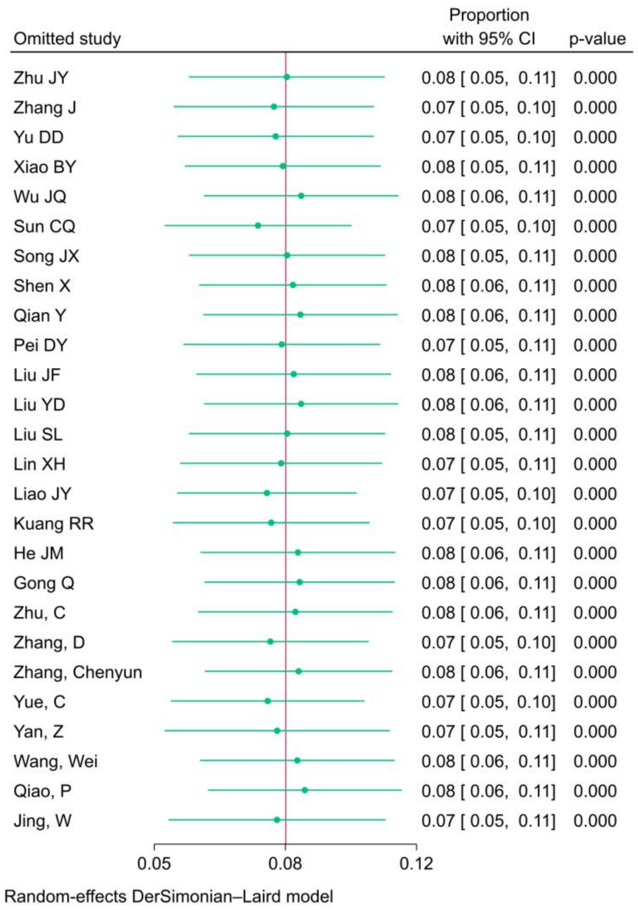

CI – confidence interval.

Figure S4. Sensitivity analysis of WHAC

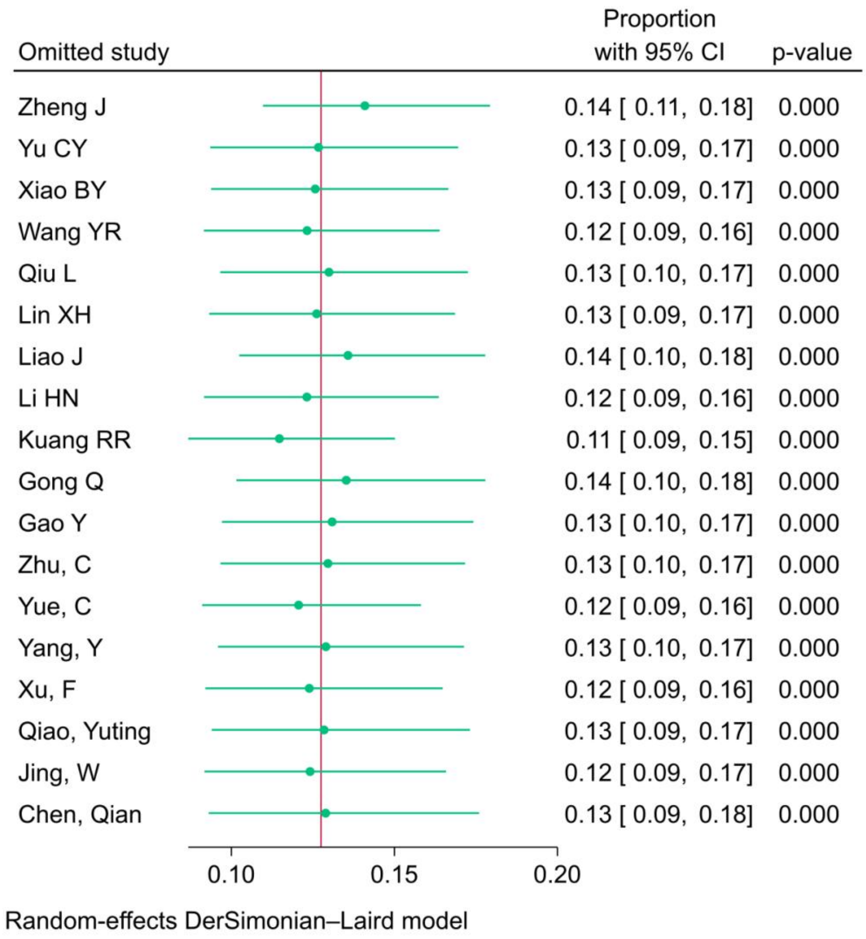

CI – confidence interval.
